# Residual Work Capacity and (In)Ability to Work Fulltime Among a Year-Cohort of Disability Benefit Applicants Diagnosed with Mental and Behavioural Disorders

**DOI:** 10.1007/s10926-023-10109-3

**Published:** 2023-03-11

**Authors:** Tialda Hoekstra, Henk-Jan Boersema, Femke I. Abma, Sandra Brouwer

**Affiliations:** 1 Department of Health Sciences, Community and Occupational Medicine, University Medical Center Groningen, University of Groningen, PO Box 196, 9700 AD Groningen, The Netherlands; 2 Research Center for Insurance Medicine (KCVG), Amsterdam, The Netherlands; 3 Dutch Social Security Institute: The Institute for Employee Benefit Schemes (UWV), Amsterdam, The Netherlands

**Keywords:** Mental and behavioural disorders, Disability benefit, Assessment, Long-term work disability

## Abstract

**Aims:**

Residual work capacity and inability to work fulltime are important outcomes in disability benefit assessment for workers with mental and behavioural disorders. The aim of this study is to gain insight into the prevalence and associations of socio-demographic and disease-related factors of these outcomes across different mental and behavioural diagnoses groups.

**Methods:**

A year cohort of anonymized register-data of patients diagnosed with a mental or behavioural disorder who claim a work disability benefit after two years of sick-leave was used (n = 12,325, age 44.5 ± 10.9, 55.5% female). Limitations in mental and physical functioning caused by disease are indicated according to the Functional Ability List (FAL). No residual work capacity was defined as having no possibilities to work, whereas inability to work fulltime was defined as being able to work less than 8 h per day.

**Results:**

The majority (77.5%) of the applicants were assessed with residual work capacity, of these 58.6% had an ability to work fulltime. Applicants diagnosed with (post-traumatic) stress, mood affective and delusional disorders showed significant higher odds for no residual work capacity and for inability to work fulltime, while other diagnoses groups, like adjustment and anxiety disorders, showed decreased odds for both assessment outcomes.

**Conclusions:**

The type of mental and behavioural disorder seems important in the assessment of residual work capacity and inability to work fulltime, as the associations differ significantly between the specific diagnoses groups.

## Introduction

Mental health-related disability poses one of the greatest social and labour market policy challenges in OECD countries. Around one-third of the annual number of new work disability benefit grants is attributable to mental and behavioural disorders [1–3] and there is a trend increase in most OECD countries [4, 5]. Besides huge economic costs at population level [4, 6], long-term disability in general and due to mental and behavioural disorders in particular, is associated at the individual level with lower socio-economic status, reduced quality of life and higher morbidity/mortality rates [7]. It is therefore of great importance to prevent the transition of short-term sickness absence into long term or permanent disability and to rehabilitate those persons already on long term disability benefit by facilitating return to work.

In the Netherlands, long-term sick-listed employees may apply for a work disability benefit after two years of sick-leave, to compensate for income loss. The insurance physician of the Dutch Social Security Institute: The Institute for Employee Benefits Schemes (UWV) assesses the health situation of an applicant and whether the applicant is able to work. When the applicant has no possibilities to perform any work at all, he or she is assessed with no residual work capacity. No residual work capacity can be assessed when an applicant is, for example, not self-reliant due to a severe mental disorder or a physical disorder [8]. When applicants are able to (partly) work, they are assessed with residual work capacity. In this latter case, the possible limitations in their mental and physical functioning caused by their disease are indicated according to the Functional Ability List (FAL) [9, 10]. This part of the assessment results in a conclusion about the (in)ability to work fulltime, reported as the number of hours the applicant can sustain working activities per day. Particularly energy deficit, fatigue and increased need for rest are primary indicators of inability to work fulltime [11, 12]. Both residual work capacity and (in)ability to work fulltime are important outcomes of work disability assessments, which usually lead to the decision of granting the benefit or not. Not only in the Netherlands, but also in many other European countries, assessing residual work capacity and (in)ability to work fulltime are part of the current work disability assessments [13, 14].

In a recent study, we showed, using register data of a year cohort of applicants assessed with residual work capacity, that the prevalence of inability to work fulltime strongly varied between different types of disease groups [15]. Moreover, we found that being diagnosed with a mental or behavioural disorder showed a significant increased risk for being assessed with inability to work fulltime compared to applicants having a disorder of another disease group. Furthermore, for applicants diagnosed with a mental or behavioural disorder, female gender and higher age were associated with an increased risk to be assessed with inability to work fulltime [15].

In our previous study we did not differentiate between the different diagnoses groups within the disease group mental and behavioural disorders as we were interested in the prevalence of (in)ability to work full-time across different disease groups [15]. However, mental and behavioural disorders include a large variety of specific diagnoses groups, like mood disorders, stress disorders and delusional disorders, which all differ in degree and patterns of work capacity impairment [16–18]. Some mental and behavioural disorders can affect self-reliance, like delusional disorders and severe addictions, while other disorders may not have such an impact. On the other hand, there are disorders that may have an impact on energy levels (e.g., mood affective disorders, schizophrenia), which may impact capacities such as endurance, while other disorders more often cause emotional disturbance (e.g., personality disorders), and impair interactional capacities (contact behaviour, group integration, assertiveness). Different qualities and patterns of capacity impairments may impact the assessment of residual work capacity and inability to work fulltime [16–18]. It can be expected that individuals having a diagnosis that comes along with a decrease in self-reliance may show increased odds for being assessed with no residual work capacity, while diagnoses associated with reduced energy levels and fatigue may show increased odds for being assessed with inability to work fulltime. On the other hand, diagnoses more associated with emotional disturbances, may have a decreased risk for being assessed with both residual work capacity and inability to work fulltime. Therefore, each mental and behavioural disorder may show a different association with residual work capacity and inability to work fulltime, and different socio-demographic and disease-related factors within each disorder may be associated with both disability assessment outcomes.

Many studies have been conducted to give more insight into the work ability description of workers with different mental and behavioural disorders. However, up to date, little is known about the prevalence of (no) residual work capacity and the (in)ability to work fulltime, two important aspects of the work disability benefit assessment in many European countries [13, 14], among workers diagnosed with a mental or behavioural disorder. Especially in employees diagnosed with these disorders, it is of great interest to distinguish between the types of diagnoses groups, since there is a large variety in the impact the different types of diagnoses have on the work capacity of these patients [16–18]. Additionally, for each diagnosis group, different socio-demographic characteristics and disease-related factors may be associated with (no) residual work capacity and (in)ability to work fulltime. Insight into these associations can contribute to a more evidence-based assessment of residual work capacity and inability to work fulltime in disability claim assessments, and may contribute to specify for which diagnoses groups supporting return to work is most useful.

Within this background, the aim of this study is to gain insight into 1) the prevalence of no residual work capacity, 2) the prevalence and degree of inability to work fulltime in case of residual work capacity, and 3) the associations of socio-demographic and disease related factors with no residual work capacity and the inability to work fulltime in a representative sample of applicants for a work disability benefit, diagnosed with a mental and behavioural disorder as their primary diagnosis, of the International Statistical Classification of Disease and Related Health Problems (ICD-10) disease group.

## Methods

### Design and Sample

The study is a cross-sectional register-based cohort study among applicants for a long-term disability benefit in the year 2016. Data were derived from the UWV register forms completed by the insurance physicians and labour experts at the time of assessment and anonymized by UWV. For this study only applicants whose primary diagnosis was a mental or behavioural disorder were included. Approval by a Medical Ethical Committee was not necessary under Dutch law, as the study is a register-based study and therefore not subject to the Medical Research Involving Human Subjects Act (WMO).

### Institutional Setting

In the Dutch social security system, workers can apply for a long-term disability benefit after two years of sick leave according to the Work and Income Act (WIA) Netherlands [19]. They may receive disability benefits for a disease or handicap due to either occupational or non-occupational causes. After a medical disability assessment by an insurance physician of the UWV, individuals can either have a full and permanent work disability, a non-permanent but full work disability, a partial work disability, or no work disability. Insurance physicians assess whether applicants have no residual work capacity if: (1) they lose their total work capacity within three months, (2) when they have a terminal disease with such a bad life expectancy that they will lose their total work capacity within foreseeable time, (3) they have fluctuating work capacity, (4) they are hospitalized, or (5) they are not self-reliant due to a severe mental disorder or a physical disorder [8]. In that case, the insurance physician can conclude to (permanent or non-permanent) full work disability. If applicants are assessed with residual work capacity, the possible limitations in their mental and physical functioning caused by their disease are indicated. After the insurance physician has completed the assessment, an additional assessment by the labour expert follows to indicate whether the applicants are incentivized to continue in paid (part-time) employment at their current employer or should enrol in a new, more appropriate (part-time) job, according to their residual work capacity.

### Measures

The presence of residual work capacity is based on the insurance physicians’ assessment (yes/no). If there is residual work capacity, the possible limitations in mental and physical functioning caused by the disease are indicated using the Functional Ability List (FAL) [9, 10]. The FAL is a standardized format list, based on the International Classification of Functioning (ICF), but with more detailed items. The 106 items of the FAL are categorized into six domains: personal functioning (30 items, e.g. focusing attention, dividing attention, insight into own abilities), social functioning (17 items, e.g. dealing with conflicts, working with others), dynamic movements (31 items, e.g. walking, use of hand and fingers), static posture (11 items, e.g. sitting at work, standing), adjusting to environment (13 items, e.g. working in an environment with dust, smoke, gases), and working hours (4 items, e.g. number of hours per day, working nights). For the current study, we used the data on the last domain, working hours, of the assessment. The number of working hours is reported by insurance physicians using 1 = at least eight hours per day; 2 = no more than eight hours per day; 3 = no more than roughly six hours per day; 4 = no more than roughly four hours per day; and 5 = no more than two hours per day. For the current study, being able to work eight or more hours per day (categories 1–2) was considered as normal ability to work fulltime, all else (categories 3–5) was considered as an inability to work fulltime.

Socio-demographic data included gender (male/female), age, and educational level. For educational level three classes were differentiated based on the highest level of completed education: low (primary school, lower vocational education, lower secondary school), middle (intermediate vocational education, upper secondary school), and high (upper vocational education, university). Educational level is usually registered by the labour expert, and therefore only part of the assessment when an applicant has residual work capacity. Consequently, educational level is often missing for applicants without residual work capacity, and therefore left out of the analyses on residual work capacity.

Insurance physicians use the Dutch Classification of Occupational Health and Social Insurance (CAS) to categorize diagnoses, derived from the International Statistical Classification of Disease and Related Health Problems (ICD-10) [20]. For generalizability, the primary, secondary and tertiary (when available) CAS-diagnoses were recoded to the 22 chapters of the ICD-10 disease groups. The type of mental and behavioural disorder was determined using the first diagnosis code. Multimorbidity was defined as having one or more additional diagnosis from a different disease group than mental and behavioural disorders.

### Statistical Methods

First, descriptive statistics were used to gain insight in the number of applicants with a primary diagnosis concerning a mental and behavioural disorder and with or without residual work capacity. Differences between applicants with and without residual work capacity were compared using t-tests for continuous data and Chi^2^-tests for categorical and ordinal data. Only specific and defined mental and behavioural disorder diagnosis groups including more than 40 applicants were included in the analyses, resulting in deleting applicants with unspecified behavioural problems, emotional sleeping disorders and unspecified mental and behavioural disorders. Second, within the group of applicants with residual work capacity and complete data on all variables, the prevalence and degree of inability to work fulltime was studied for the total group and for each specific mental health diagnosis group. Third, univariable and multivariable logistic regression analyses were performed to study the association of each socio-demographic variable (age, gender) and disease related variable (multimorbidity) with no residual work capacity (no/yes). Fourth, univariable and multivariable logistic regression analyses (adjusted for age, gender, multimorbidity, for the analyses on residual work capacity, educational level was added for the analyses on inability to work fulltime) were performed to study the association of the specific mental and behavioural disorder diagnosis groups with no residual work capacity and inability to work fulltime. Fifth, multivariable logistic regression analyses were performed, stratified to the mental and behavioural disorder diagnosis groups, to study the association of each socio-demographic variable (age, gender for no residual work capacity and additionally educational level for inability to work fulltime) and disease-related variable (multimorbidity) with no residual work capacity and inability to work fulltime within the specific mental health diagnosis groups.

Analyses were performed using IBM SPSS Statistics version 25. For all analyses a p-level of < 0.05 was considered to indicate statistical significance.

## Results

**Fig. 1:**
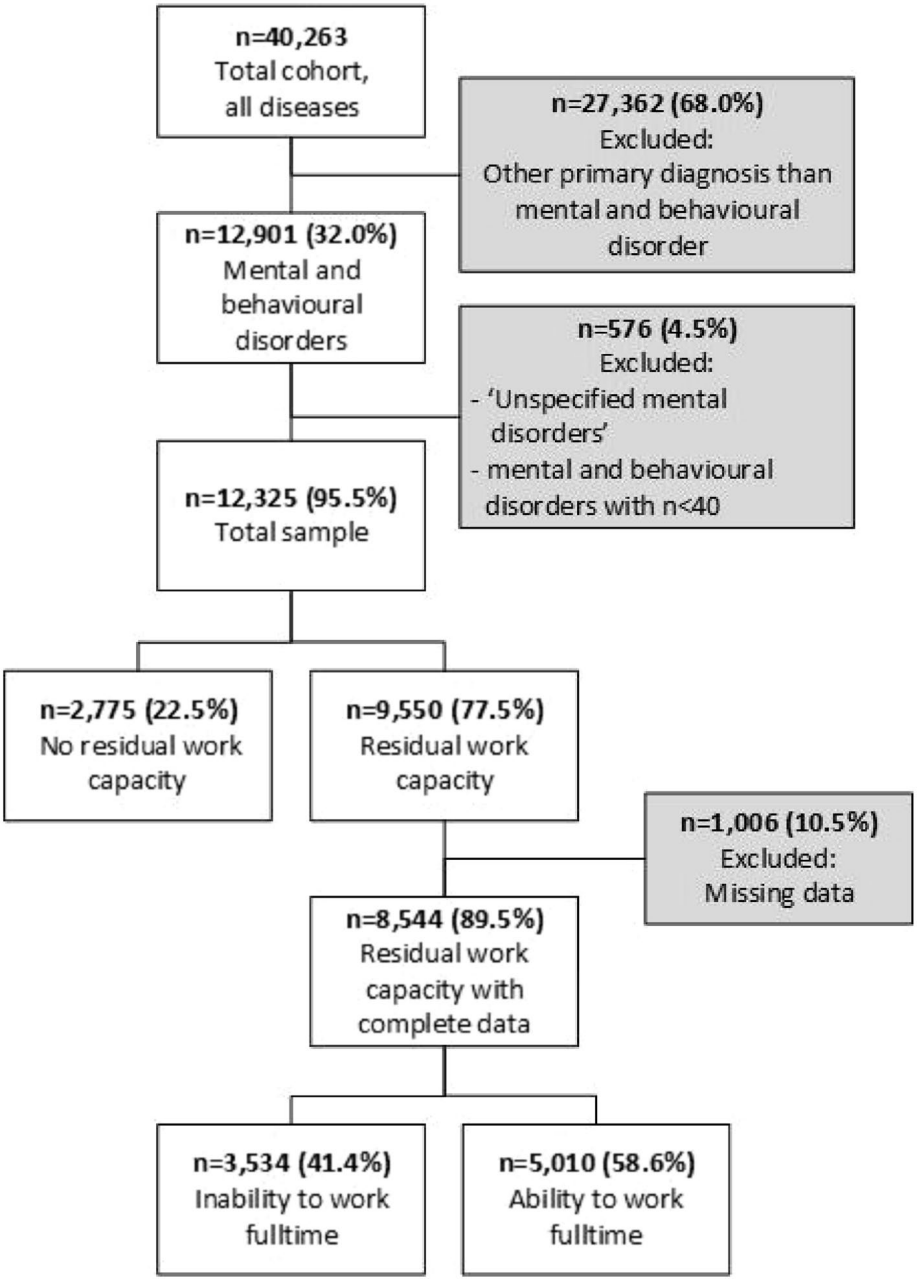
Overview of the inclusion flow

Data from 40,263 applicants for a WIA benefit in 2016 (mean age 48.7 (± 11.0) years; 53.6% women) were used. Of these, 12,901 (32.0%) had a mental or behavioural disorder as the primary diagnosis (mean age 44.4 (± 11.0) years; 55.4% women). After removal of applicants with unspecified mental disorders and diagnoses groups with 40 or less applicants, the dataset included 12,325 disability benefit applicants with a mental or behavioural disorder (Fig. 1).

### No Residual Work Capacity

Of the 12,325 applicants, 77.5% (n = 9550) were assessed with residual work capacity. Applicants without residual work capacity were younger, more often male and had less often multimorbidity than applicants with residual work capacity (see Table 1). Educational level was difficult to compare due to a high percentage of missing data, especially in the group without residual work capacity. Applicants diagnosed with (post-traumatic) stress disorders, mood affective disorders, addictions, and schizophrenia and delusional disorders were significantly more present in the group with no residual work capacity, while applicants diagnosed with mental retardation, autism spectrum disorders, ADHD, somatoform disorders, adjustment disorders (including burn-out), and anxiety disorders were significantly more present in the group assessed with residual work capacity (Table 1).

**Table 1:** Characteristics and differences between work disability benefit applicants regarding residual work capacity and ability to work fulltime

	Total group (n = 12,325) N (%)	No residual work capacity (N = 2775, 22.5%) N (%)	Residual work capacity (N = 9550, 77.5%) N (%)	p-value	Total group (n = 8544) N (%)	Inability to work fulltime (N = 3534, 41.4%) N (%)	Ability to work fulltime (N = 5010, 58.6%) N (%)	p-value
Age (years) (mean ± SD)	44.5 ± 10.9	43.4 ± 10.7	44.8 ± 10.9	< .001	44.8 ± 11.0	45.0 ± 11.0	44.8 ± 10.9	.538
Female gender	6815 (55.5%)	1485 (53.5%)	5330 (55.8%)	.032	4815 (56.4%)	2194 (62.1%)	2621 (52.3%)	< .001
Educational level^a^				< .001				.117
Low	4155 (44.4%)	430 (53.1%)	3725 (43.6%)		3725 (43.6%)	1510 (42.7%)	2215 (44.2%)	
Middle	3279 (35.1%)	274 (33.8%)	3005 (35.2%		3005 (35.2%)	1288 (36.4%)	1717 (34.3%)	
High	1920 (20.5%)	106 (13.1%)	1814 (21.2%)		1814 (21.2%)	736 (20.8%)	1078 (21.5%)	
Multimorbidity	4916 (39.9%)	788 (28.4%)	4128 (43.2%)	< .001	3823 (44.7%)	1579 (44.7%)	2244 (44.8%)	.920
Degree of ability to work fulltime								< .001
> 8 h per day					3941 (46.1%)	–	3941 (78.7%)	
≤ 8 h per day					1069 (12.5%)	–	1069 (21.3%)	
≤ 6 h per day					732 (8.6%)	732 (20.7%)	–	
≤ 4 h per day					2285 (26.7%)	2285 (64.7%)	–	
≤ 2 h per day					517 (6.1%)	517 (14.6%)	–	
Mental and behavioural disorder								
Mental retardation	236 (1.9%)	35 (1.3%)	201 (2.1%)	.004	124 (1.4%)	39 (1.1%)	84 (1.7%)	.029
Autism spectrum disorders	427 (3.5%)	62 (2.2%)	365 (3.8%)	< .001	310 (3.6%)	116 (3.3%)	194 (3.9%)	.151
ADHD	281 (2.3%)	22 (0.8%)	259 (2.7%)	< .001	229 (2.7%)	76 (2.2%)	153 (3.1%)	.011
Somatoform disorders	495 (4.0%)	75 (2.7%)	420 (4.4%)	< .001	388 (4.5%)	155 (4.4%)	233 (4.7%)	.563
Adjustment disorders (including burn-out)	1402 (11.4%)	74 (2.7%)	1328 (13.9%)	< .001	1201 (14.1%)	323 (9.1%)	878 (17.5%)	< .001
(Post-traumatic) stress disorders	1743 (14.1%)	475 (17.1%)	1268 (13.3%)	< .001	1125 (13.2%)	531 (15.0%)	594 (11.9%)	< .001
Anxiety disorders	1019 (8.3%)	158 (5.7%)	861 (9.0%)	< .001	774 (9.1%)	278 (7.9%)	496 (9.9%)	.001
Personality disorders	901 (7.3%)	211 (7.6%)	690 (7.2%)	.500	626 (7.3%)	215 (6.1%)	411 (8.2%)	< .001
Mood affective disorders	4894 (39.7%)	1277 (46.0%)	3617 (37.9%)	< .001	3318 (38.8%)	1585 (44.9%)	1733 (34.6%)	< .001
Addictions	418 (3.4%)	158 (5.7%)	260 (2.7%)	< .001	235 (2.8%)	78 (2.2%)	157 (3.1%)	.010
Schizophrenia and delusional disorders	509 (4.1%)	228 (8.2%)	281 (2.9%)	< .001	215 (2.5%)	138 (3.9%)	77 (1.5%)	< .001

^a^Frequencies do not add up to the total n due to missing values

### Inability to Work Fulltime

Of the 9,550 applicants with residual work capacity, 8544 (89.5%) applicants had complete data on all variables. Of the applicants with missing data (n = 1006, mainly on educational level), the majority (67.4%) had a normal ability to work fulltime, whereas in the study sample, including applicants with complete data, 58.6% had normal ability to work fulltime (p < 0.001).

Of the applicants assessed with an inability to work fulltime, the majority (64.7%) were considered to be able to work about four hours per day (Table 1). Applicants with an inability to work fulltime were significantly more often female. Age, educational level and multimorbidity did not differ significantly between applicants with an ability and an inability to work fulltime. Applicants diagnosed with (post-traumatic) stress disorders, mood affective disorders, and schizophrenia and delusional disorders were significantly more present in the group assessed with an inability to work fulltime, while applicants diagnosed with mental retardation, ADHD, adjustment disorders (including burn-out), anxiety disorders, personality disorders and addictions were significantly more present in the group assessed with an ability to work fulltime (Table 1).

### Associations with No Residual Work Capacity and Inability to Work Fulltime

Age, gender and multimorbidity were significantly associated with no residual work capacity in the multivariable analyses, where higher age, female gender and being diagnosed with an additional disorder resulted in lower odds for no residual work capacity (Table 2).

**Table 2:** Associations of socio-demographic and disease related variables with no residual work capacity (univariable and multivariable logistic regression analyses)

	No residual work capacity (n = 12,325)					
	Univariable analyses			Multivariable analyses		
	OR	95% CI	p-value	OR	95% CI	p-value
Age (years)	0.99	0.98–0.99	< .001	0.99	0.99–1.00	.001
Female gender	0.91	0.84–0.99	.032	0.91	0.83–0.99	.028
Multimorbidity	0.52	0.48–0.57	< .001	0.54	0.49–.59	< .001

Of the specific diagnoses groups, (post-traumatic) stress disorders, mood affective disorders, addictions and schizophrenia and delusional disorders showed significant higher odds for no residual work capacity, both in univariable and multivariable regression analyses. On the other hand, mental retardation, autism spectrum disorders, ADHD, somatoform disorders, adjustment disorders (including burn-out), and anxiety disorders showed significant lower odds for no residual work capacity. Of all mental and behavioural disorders, only the diagnosis group personality disorders was not associated with no residual work capacity (Table 3).

**Table 3:** Associations of the mental and behavioural disorder diagnosis groups with no residual work capacity and inability to work fulltime (univariable and multivariable logistic regression analyses, adjusted for age, gender, educational level (only analyses on inability to work fulltime) and multimorbidity)

	No residual work capacity (n = 12,325)						Inability to work fulltime (n = 8,544)					
	Univariable analyses			Multivariable analyses			Univariable analyses			Multivariable analyses		
	OR	95% CI	p-value	OR	95% CI	p-value	OR	95% CI	p-value	OR	95% CI	p-value
Mental retardation	0.59	0.41–0.85	.005	0.62	0.43–0.90	.011	0.65	0.45–0.96	0.030	0.70	0.48–1.03	0.072
Autism spectrum disorders	0.58	0.44–0.76	< .001	0.51	0.39–0.67	< .001	0.84	0.67–1.07	0.151	0.96	0.76–1.22	0.729
ADHD	0.29	0.19–0.44	< .001	0.26	0.17–0.40	< .001	0.70	0.53–0.92	0.011	0.76	0.57–1.01	0.055
Somatoform disorders	0.60	0.47–0.78	< .001	0.62	0.48–0.80	< .001	0.94	0.76–1.16	0.563	0.90	0.73–1.11	0.309
Adjustment disorders (including burn-out)	0.17	0.13–0.22	< .001	0.18	0.14–0.23	< .001	0.47	0.41–0.54	< .001	0.44	0.38–0.51	< .001
(Post-traumatic) stress disorders	1.35	1.20–1.51	< .001	1.36	1.21–1.52	< .001	1.32	1.16–1.49	< .001	1.30	1.14–1.48	< .001
Anxiety disorders	0.61	0.51–0.73	< .001	0.58	0.49–0.69	< .001	0.78	0.67–0.91	.001	0.76	0.65–0.89	.001
Personality disorders	1.06	0.90–1.24	.500	0.98	0.84–1.16	.840	0.73	0.61–0.86	< .001	0.71	0.60–0.85	< .001
Mood affective disorders	1.40	1.28–1.52	< .001	1.50	1.37–1.63	< .001	1.54	1.41–1.68	< .001	1.54	1.41–1.68	< .001
Addictions	2.16	1.76–2.64	< .001	2.05	1.67–2.52	< .001	0.70	0.53–0.92	0.010	0.78	0.60–1.05	.105
Schizophrenia and delusional disorders	2.95	2.47–3.54	< .001	2.56	2.13–3.08	< .001	2.60	1.96–3.45	< .001	3.02	2.26–4.02	< .001

With regards to inability to work fulltime, (post-traumatic) stress disorders, mood affective disorders and schizophrenia and delusional disorders showed significant higher odds for the inability to work fulltime, whereas adjustment disorders (including burn-out), anxiety disorders and personality disorders showed significant lower odds for being assessed with an inability to work fulltime (Table 3).

### Associations with No Residual Work Capacity and Inability to Work Fulltime Within Specific Mental Health Diagnosis Groups

The multivariable logistic regression analyses, stratified to the specific mental and behavioural disorder diagnoses groups, showed that for applicants with a (post-traumatic) stress disorder, women had lower odds to be assessed with no residual work capacity. For applicants with a somatoform disorder or an anxiety disorder, a higher age was negatively associated with no residual work capacity. Multimorbidity was negatively associated with no residual work capacity for applicants with autism spectrum disorders, (post-traumatic) stress disorders, anxiety disorders, personality disorders, mood affective disorders, addictions, or schizophrenia and delusional disorders (Table 4).

**Table 4:** Associations of gender, age, and multimorbidity with no residual work capacity stratified to the mental and behavioural disorder diagnosis groups (multivariable logistic regression analyses)

	Gender (male = ref) OR (95%CI)	Age OR (95% CI)	Multimorbidity OR (95%CI)
Mental retardation	0.94 (0.44–1.99)	1.00 (0.97–1.04)	0.86 (0.41–1.81)
Autism spectrum disorders	0.85 (0.46–1.57)	0.99 (0.96–1.01)	0.49 (0.25–0.96)*
ADHD	0.78 (0.31–1.99)	1.00 (0.95–1.05)	0.52 (0.18–1.50)
Somatoform disorders	1.21 (0.68–2.13)	0.96 (0.94–0.99)*	1.21 (0.73–2.01)
Adjustment disorders (including burn-out)	1.10 (0.69–1.81)	1.00 (0.97–1.02)	1.15 (0.72–1.86)
(Post-traumatic) stress disorders	0.79 (0.63–0.98)*	1.00 (0.99–1.01)	0.51 (0.41–0.65)*
Anxiety disorders	1.18 (0.82–1.70)	0.98 (0.96–0.99)*	0.56 (0.38–0.83)*
Personality disorders	1.11 (0.80–1.55)	0.98 (0.97–1.00)	0.44 (0.30–0.65)*
Mood affective disorders	1.06 (0.93–1.21)	1.00 (0.99–1.00)	0.53 (0.46–0.61)*
Addictions	0.72 (0.43–1.19)	0.99 (0.97–1.01)	0.47 (0.29–0.75)*
Schizophrenia and delusional disorders	0.89 (0.60–1.31)	1.01 (0.99–1.03)	0.47 (0.29–0.77)*

*p < .05

The stratified analyses for inability to work fulltime, showed that for applicants with mental retardation or a mood affective disorder, higher age was associated with an increased odds for inability to work fulltime. Whereas for applicants with ADHD, adjustment disorders (including burn-out), (post-traumatic) stress disorders, personality disorders, mood affective disorders, addictions, and schizophrenia and delusional disorders, female gender was significantly associated with higher odds for inability to work fulltime. A middle educational level (compared to a low educational level) showed increased odds for inability to work fulltime for applicants with mental retardation or a somatoform disorder, and a high educational level was associated with inability to work fulltime within applicants with a personality disorder. Multimorbidity was negatively associated with inability to work fulltime within applicants with an autism spectrum disorder (Table 5).

**Table 5:** Associations of gender, age, educational level and multimorbidity with the inability to work fulltime stratified to the mental and behavioural disorder diagnosis (multivariable logistic regression analyses)

	Gender (male = ref) OR (95%CI)	Age OR (95% CI)	Educational level (low = ref)		Multimorbidity OR (95%CI)
			Middle OR (95%CI)	High OR (95%CI)	
Mental retardation	1.83 (0.77–4.36)	1.06 (1.03–1.10)*	7.03 (1.04–47.32)*	–	1.32(0.53–3.29)
Autism spectrum disorders	1.63 (0.97–2.73)	1.01 (0.99–1.04)	1.74 (0.99–3.05)	1.14 (0.60–2.15)	0.56 (0.34–0.94)*
ADHD	2.52 (1.34–4.74)*	0.98 (0.95–1.01)	1.68 (0.89–3.18)	0.60 (0.24–1.52)	1.36 (0.74–2.48)
Somatoform disorders	1.14 (0.72–1.81)	1.00 (0.98–1.02)	2.05 (1.25–3.38)*	1.28 (0.75–2.19)	1.13 (0.74–1.74)
Adjustment disorders (including burn-out)	2.11 (1.57–2.82)**	1.01 (0.99–1.02)	1.27 (0.92–1.75)	1.08 (0.77–1.51)	1.21 (0.92–1.59)
(Post-traumatic) stress disorders	1.74 (1.35–2.24)**	1.00 (0.99–1.01)	1.08 (0.83–1.40)	0.92 (0.65–1.31)	0.93 (0.72–1.18)
Anxiety disorders	1.36 (1.00–1.86)	1.01 (0.99–1.02)	0.86 (0.62–1.20)	0.77 (0.51–1.17)	1.01 (0.74–1.37)
Personality disorders	1.47 (1.02–2.10)*	1.00 (0.99–1.02)	1.20 (0.81–1.78)	1.86 (1.19–2.90)*	0.87 (0.61–1.25)
Mood affective disorders	1.51 (1.31–1.74)**	1.01 (1.00–1.01)*	0.98 (0.84–1.15)	0.96 (0.80–1.16)	0.94 (0.81–1.08)
Addictions	1.89 (1.02–3.52)*	1.00 (0.97–1.03)	1.29 (0.72–2.32)	0.94 (0.27–3.32)	1.51 (0.82–2.76)
Schizophrenia and delusional disorders	2.61 (1.31–5.23)*	0.98 (0.95–1.01)	0.83 (0.44–1.57)	1.51 (0.63–3.63)	0.69 (0.35–1.40)

*p < .05, **p < .001

## Discussion

The findings of our study are in line with our expectations. Especially the diagnoses groups that are associated with a decreased self-reliance (e.g., (post-traumatic) stress disorders, mood affective disorders, schizophrenia and delusional disorders), are associated with increased odds for no residual work capacity. These diagnoses are known to affect the energy levels as well, resulting in increased odds for inability to work fulltime, when there was residual work capacity. On the other hand, diagnoses that affect energy levels less (e.g., ADHD, somatoform disorders) or that are related with emotional disturbances (e.g., personality disorders), showed decreased odds for being assessed with inability to work fulltime. We conducted a similar study regarding applicants diagnosed with cancer as the primary diagnosis [21]. Although when being diagnosed with cancer, other factors, like survival rate, play a role. Our results, indeed, showed that cancers with a low survival rate (like respiratory cancers) were associated with no residual work capacity. However, with regards to being assessed with inability to work fulltime, the results are comparable. Especially cancers that have a negative impact on energy levels (lymphoid and haematopoietic cancers, and cancers of the respiratory organs) showed increased odds for inability to work fulltime [21]. This might not be surprising, as energy deficit and fatigue are mentioned as the primary indicators of inability to work fulltime [11, 12].

Other mental and behavioural disorders, like mental retardation, autism spectrum disorders, ADHD, somatoform disorders, adjustment disorders (including burn-out), and anxiety disorders showed decreased odds for being assessed with no residual work capacity. Additionally, for adjustment, anxiety and personality disorders we found decreased odds for being assessed with inability to work fulltime. This confirms the high variety among mental and behavioural disorders with regards to the ability to work. In other words, diagnosis matters. For mental retardation and developmental disorders like autism spectrum disorders and ADHD, these results may seem surprising, as the employment rates of individuals with these disorders are very low [22–25]. It is therefore important to realize that our study population concerns individuals who were employed and on sick leave for about 2 years. In the Netherlands young adults with congenital disabilities or disabilities originated during childhood (before the age of 18) can apply for a disability benefit based on ‘Invalidity Insurance Act for Young Disabled Persons’ (Wajong Act) [26]. As the current sample was already active on the labour market, it is quite possible that insurance physicians are less inclined to assess them with no residual work capacity.

For addiction the results seem counterintuitive, as there is an increased risk for being assessed with no residual work capacity, but a decreased risk for being assessed with an inability to work fulltime. An explanation for this result could be that the severe cases are admitted to rehabilitation clinics at the time of assessment, and therefore have no residual work capacity. However, the less severe patients, and the patients who are not admitted (anymore) to a clinic, should be able to work fulltime according to the insurance physician. Having an addiction is seen as a chronic condition, but once in remission, does not seem to impact the ability to work in a way that an inability to work fulltime is indicated [27–29].

A notable finding is the decreased odds of multimorbidity for being assessed with no residual work capacity within most of the diagnoses groups. The association of being diagnosed with more than one disease seems counterintuitive, because one could expect that this would have an increased impact on work ability. However, we also found this result in our study on residual work capacity and inability to work fulltime within cancer patients [21]. We discussed these findings with insurance physicians, and they thought a possible explanation might be that when the primary diagnosis is so severe and has a major impact on work capacity, they feel further explanation of the medical situation is unnecessary. In these cases, they do not register any additional diagnoses.

### Strengths and Limitations

In this study we used register-data of a year cohort of applicants assessed for a work disability benefit after 2 years of sick leave. Using register-data is a strength of our study, as it covers the entire Dutch population including data on socio-demographic variables and all mental and behavioural diagnoses. This gave us the opportunity to compare the work disability assessment outcomes of the specific diagnoses groups. Another strength of our study is the large sample size of work disability benefit assessments by skilled insurance physicians adhering to professional guidelines and assessment methods. On the other hand, using register-data is also a limitation to our study, as the data was not collected for research purposes and therefore information on the severity of the disorder, treatment and personal factors are not available. Furthermore, for the analyses on inability to work fulltime, we had to exclude 1,006 cases due to missing data mostly on educational level. This might have impacted our outcomes, as the prevalence of being assessed with a normal ability to work fulltime was higher among the excluded sample than in the selected sample. Furthermore, because of the cross-sectional design, we are not able to draw conclusions on causal relationships.

### Implications for Practice and Future Research

The findings of our study show that the majority of the applicants with mental and behavioural disorders for a work disability benefit have residual work capacity and are assessed with a normal ability to work fulltime. This implies that (supporting) return to work is of great importance among individuals with mental and behavioural disorders who are on sick-leave as the chances of receiving a work disability benefit, two years after sick-leave, are low. As the disease group ‘mental and behavioural disorders’ concerns a wide variety of diseases, including a wide variety in the effect on self-reliance, energy levels and emotion regulation, there are large differences between the diagnoses groups for the odds of being assessed with residual work capacity or inability to work fulltime. Applicants of the different diagnoses groups might therefore require a different approach with regards to the assessment and the support for return to work. Our study contributes to providing insight into for which specific diagnoses groups supporting return to work is most useful. Furthermore, our findings can contribute to a more evidence-based assessment of residual work capacity and inability to work fulltime in disability claim assessments, providing insight into which workers within mental and behavioural disorder diagnoses groups are at risk for no residual work capacity and inability to work fulltime.

Our study aimed to explore two important work outcomes of the disability benefit assessment, using register data from the UWV. Future research including other indicators like the individual diagnosis, the severity of the disease, treatment, work limitations and other personal and environmental factors, could provide more insight in possible indicators for no residual work capacity and inability to work fulltime and a clearer understanding of work (dis)ability phenomenology. Additionally, longitudinal studies should be conducted on the work trajectories from the onset of sick leave until after the disability assessment of patients diagnosed with different types mental and behavioural disorders. These studies will provide insight into the possible changes in ability to work of individuals with mental and behavioural disorders before and after the disability benefit assessment. It will also provide insight on the effect of being assessed with (in)ability to work fulltime on actual (return to) work after the assessment.

## Conclusion

Our results showed that among work disability benefit applicants with a mental or behavioural disorder, about three quarters are assessed with residual work capacity, and of these, the majority is assessed with a normal ability to work fulltime, two years after sick leave. However, the type of mental and behavioural disorder seems important in terms of the assessment of residual work capacity and the ability to work fulltime, as the associations with these outcomes differ significantly between the specific diagnoses groups. The findings of our study can contribute to a more evidence-based assessment of residual work capacity and inability to work fulltime in disability claim assessments, providing insight into which workers within specific diagnoses groups are at risk for both outcomes. Subsequently, our study provides insight into which workers within specific diagnoses groups are not at risk for both outcomes, and might benefit from additional support to improve return to work.

## Data Availability

The data that support the findings of this study were made available from UWV. However, restrictions apply to the availability of these data, which were used under license for the current study, and are not publicly available. Data are however available from the authors upon reasonable request and with permission of UWV.
